# Theoretical Studies of Anisotropic Melting of Ice Induced by Ultrafast Nonthermal Heating

**DOI:** 10.1021/acsphyschemau.3c00072

**Published:** 2024-05-08

**Authors:** Ibrahim Dawod, Kajwal Patra, Sebastian Cardoch, H. Olof Jönsson, Jonas A. Sellberg, Andrew V. Martin, Jack Binns, Oscar Grånäs, Adrian P. Mancuso, Carl Caleman, Nicusor Timneanu

**Affiliations:** ‡ Department of Physics and Astronomy, Uppsala University, Box 516, SE-751 20 Uppsala, Sweden; ¶ European XFEL, Holzkoppel 4, DE-22869 Schenefeld, Germany; § Department of Applied Physics, KTH Royal Institute of Technology, SE-106 91 Stockholm, Sweden; ∥ School of Science, RMIT University, Melbourne, Victoria 3000, Australia; ⊥ Diamond Light Source, Harwell Science and Innovation Campus, Didcot OX11 0DE, U.K.; # Department of Chemistry and Physics, La Trobe Institute for Molecular Science, La Trobe University, Melbourne, Victoria 3086, Australia; ∇ Center for Free-Electron Laser Science, Deutsches Elektronen-Synchrotron, Notkestraße 85, DE-22607 Hamburg, Germany

**Keywords:** X-ray free-electron laser, ultrafast dynamics, nonthermal melting, molecular dynamics, plasma simulations, coherent diffractive imaging

## Abstract

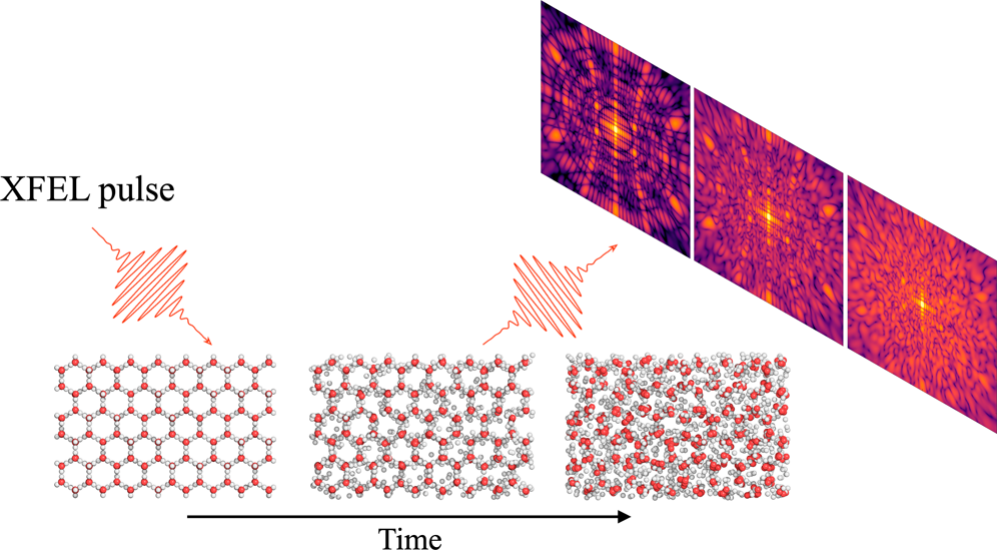

Water and ice are routinely studied with X-rays to reveal their diverse structures and anomalous properties. We employ a hybrid collisional-radiative/molecular-dynamics method to explore how femtosecond X-ray pulses interact with hexagonal ice. We find that ice makes a phase transition into a crystalline plasma where its initial structure is maintained up to tens of femtoseconds. The ultrafast melting process occurs anisotropically, where different geometric configurations of the structure melt on different time scales. The transient state and anisotropic melting of crystals can be captured by X-ray diffraction, which impacts any study of crystalline structures probed by femtosecond X-ray lasers.

## Introduction

Water has a rich phase diagram with at least ten thermodynamically stable crystalline phases that have been isolated and characterized.^1^ Many of the crystal polymorphs are topologically tetrahedral, meaning that each water molecule forms four hydrogen bonds to its nearest neighbors. This creates a local tetrahedral structure with a second hydration shell occurring at  of the first shell’s distance. On Earth, the most common form of ice is hexagonal ice (*I*_*h*_), with a nearest neighbor distance of 2.75 Å.^2^ When hexagonal ice melts, the periodicity of the crystalline lattice is lost, but the local tetrahedral structure is to a large extent retained, while the nearest neighbor distance increases slightly to 2.80 Å.^3^ Melting of ice occurs by exciting vibrational modes of the water molecules, which causes local disorder that later spreads in the crystal.^4^ If the energy is deposited by an ultrafast infrared laser, superheated states can emerge where the crystal reaches temperatures above melting, but the crystalline structure stays intact for picoseconds.^4,5^ Recent experiments using the Linac Coherent Light Source (LCLS) in Stanford have demonstrated a fundamentally different pathway to heat water, namely through intense ionization, so-called nonthermal heating.^6^ Water is exposed to sub-100 fs intense X-ray pulses, which heats it above 100 000 K, driving the liquid into a plasma state. The experimental observations were understood using a combination of collisional radiative (CR) simulations under a non-local thermodynamic equilibrium (NLTE) condition and molecular dynamics (MD) simulations. With an updated physical model where the CR calculations are used to dynamically change the force field in the classical MD simulations, our method can now show that under an intense X-ray pulse we force ice into a yet unexplored phase of water, namely that of a crystalline plasma. Plasma crystals have been described before for low pressure one-component plasma systems consisting of single charged particles in a uniform neutralizing background charge.^7−9^ What we describe here is instead a rarely studied phase of matter, a solid density plasma-ice, only reached using the intense X-ray bombardment from an XFEL source. Studying different phases of water using an XFEL source is important due to their appearance in biological systems such as protein crystals, which nowadays are routinely studied using serial femtosecond crystallography (SFX).^10,11^

As the water molecules in ice are exposed to the X-rays, processes such as photoabsorption and secondary ionization from free electrons causes the molecules to be become charged. This leads to bond breaking and a phase transition from the solid phase direct to the plasma state.^6^ On femtosecond time scales the increasing free electron density and temperature is expected to trigger Debye shielding that reduces the Coulomb interaction between the ions and preserves the crystalline structure longer. As the free-electron background gas is expected to be uniform and bond breaking results in forces being spherically symmetric, the lattice anisotropy would dictate the dynamics of the melting.^12^ Beyond femtosecond time scales the sample is deeply into a plasma regime and will eventually evaporate.

**Figure 1:**
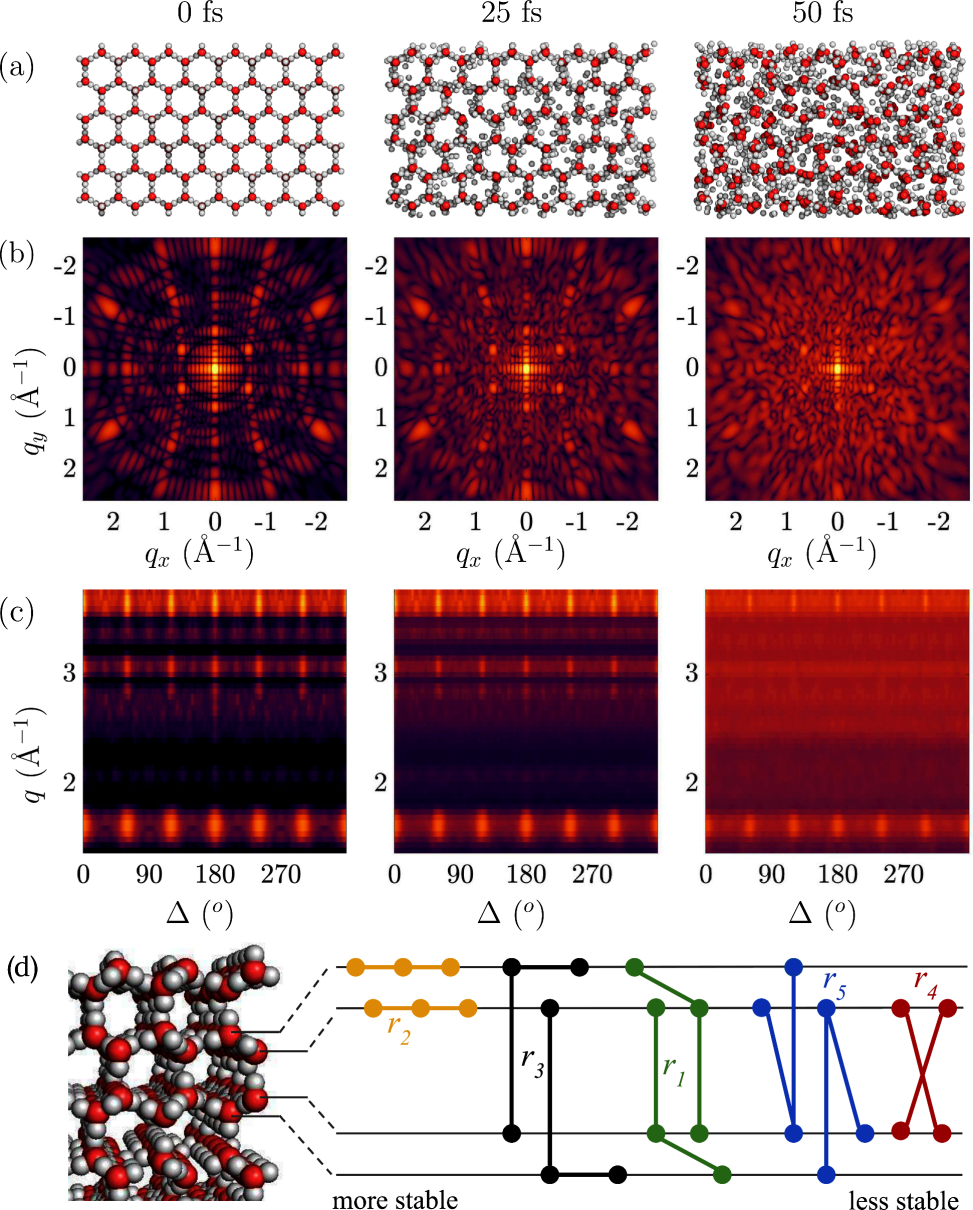
Dynamics of hexagonal ice investigated with an intense 50 fs long X-ray pulse, with 8 keV photons and intensity of 1 × 10^18^ W cm^–2^. (a) Snapshots of the structures from the hybrid CR/MD simulation of ice at 3 different time points. (b) Diffraction patterns of ice at corresponding time points, with sample to detector distance of 70 mm. (c) Angular correlation of the intensity in the diffraction patterns as a function of angle and momentum transfer. (d) Side view of the ice structure with schematic of the hexagonal planes and angular pairs of oxygen atoms. It visualizes the planes the 3- and 4-body terms occupy. This affects how long these structures persist (their degree of stability on the fs time-scale) when exposed to the XFEL.

Given the right conditions, this exotic state of crystalline plasma can exist for multiples of ten femtoseconds. According to our calculations this state should be experimentally possible to observe in single shot diffraction experiments using femtosecond X-ray sources such as European XFEL (EuXFEL) or the Linac Coherent Light Source (LCLS). Figure 1 captures the melting of hexagonal ice on femtosecond time scales and gives an interplay of our theoretical study with a possible experiment. In this study we follow the ultrafast dynamics in the real space (panel a). A diffraction experiment would capture the sample structure in the reciprocal space. Here we simulate X-ray diffraction snapshots on an area detector (panel b), based on each atom’s position and electronic configuration. Our model accounts for the fact that the form-factor of each atom in the system can be different due to ionization states induced by the pulse. An angular correlation analysis of the patterns (panel c) could be used to investigate melting in the reciprocal space, and this can be directly compared with single shot experiments. We visualize here one particular orientation of a finite size crystal, and therefore some reflections are missing due to the curvature of the Ewald sphere (which depends on the photon energy and detector to sample distance) and size artifacts will occur. Finally, using the real space information from our simulations, we investigate dynamics of the correlations through pair angle distributions. A sketch of the 3-point and 4-point pairs of the oxygen atoms in the different hexagonal planes show which pairs keep correlated longer during the 50 fs pulse (panel d).

## Results and Discussion

We focus our investigation on the stability of different atomic distances and angles of the crystalline structure of ice aiming to identify those that remain the longest into the plasma state. We developed a two-step hybrid collisional radiative (CR)/molecular dynamics (MD) model^13^ where the CR simulations are used to update continuously the force field in the MD simulations. From these, we can follow charge dynamics and real space positions of the atoms in time. Detailed information about the model can be located in the method section. Our simulations are done using experimentally relevant beam parameters that are achievable at XFELs. We simulated a system exposed to a 50 fs flat top shaped pulse with intensities ranging between 10^18^ – 10^19^ Wcm^–2^ and 8 keV energy photons, similar to parameters used in earlier studies.^6^ We note that we simulate an idealized X-ray pulse, while the experimental pulses might be spiky and this could influence the ionization dynamics, but will have a limited effect on the atomic dynamics. Results using the lowest intensity (10^18^ Wcm^–2^) are shown here and some of the results for higher intensities (5 × 10^18^ and 10^19^ Wcm^–2^) are presented in the Supporting Information (SI).

**Figure 2:**
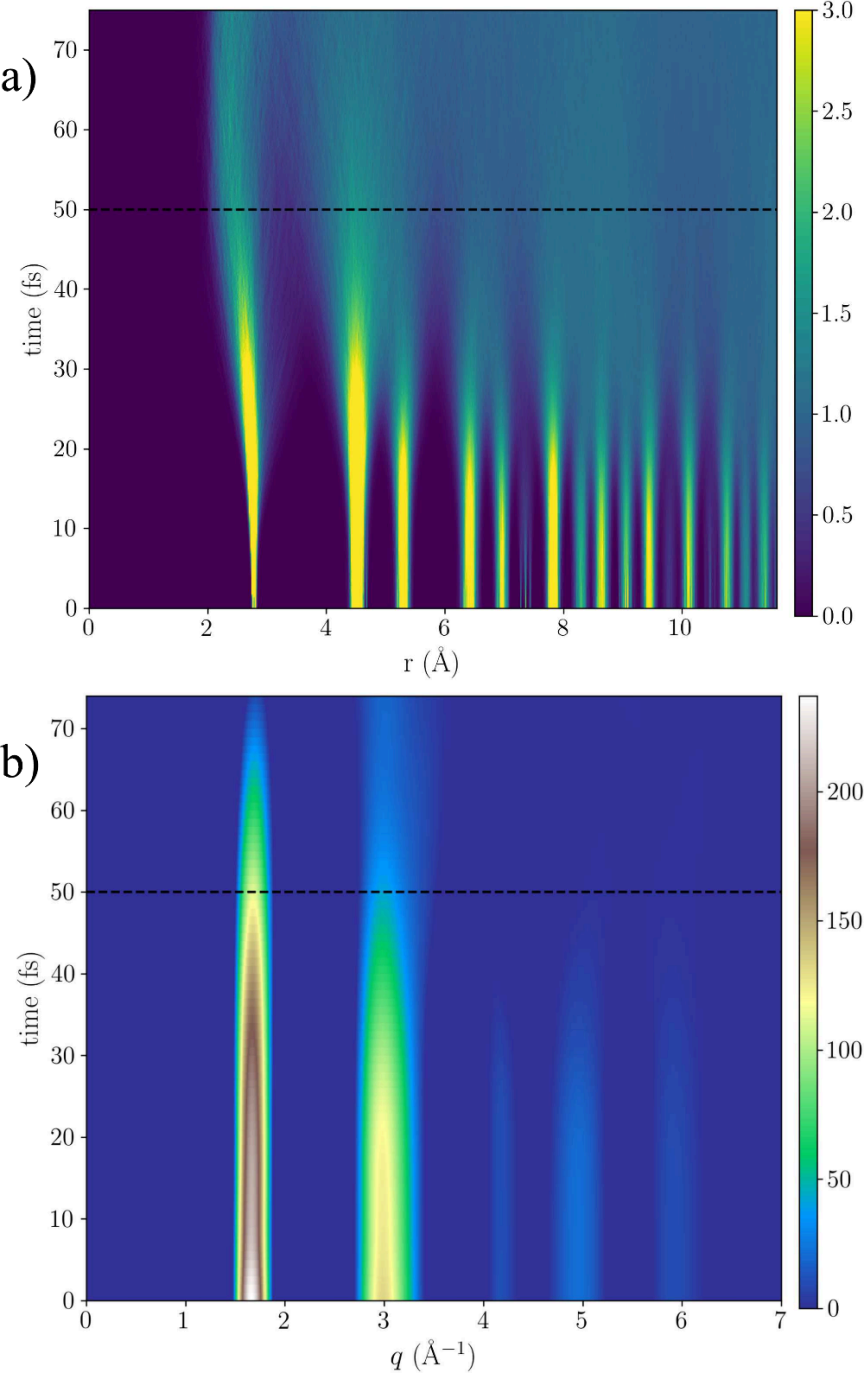
(a) Time evolution of the radial distribution function for O–O distances in ice, as a result of a 50 fs long X-ray (dashed line) with an intensity of 10^18^ W cm^–2^ and photon energy of 8 keV. (b) Time evolution of the intermolecular intensity *I*_*inter*_(*q*, *t*) = *∑*_α,β_(2 – δ_α,β_)*c*_α_*c*_β_*f*_α_(*q*, *t*)*f*_β_(*q*, *t*)*S*_α,β_(*q*, *t*), which includes contribution from atomic displacement *S*_α,β_ and changes in the form-factors *f*_α_, *f*_β_ due to electronic damage, summed over all fractional populations of ions *c*_α_, *c*_β_. Scattering due the hydrogen atoms contributes weakly and is shown explicitly in the SI. The results in both figures are averaged over multiple trajectories with different charge distributions. The color scale is in arbitrary units.

From the time dependent oxygen–oxygen (O–O) radial distribution function (RDF) in Figure 2 we can study the probability of finding two oxygen atoms at particular distances. In the initial part of the pulse, the RDF shows discrete peaks due to the repeating structure of the atoms in the crystal. As time evolves, the periodicity is lost and the RDF becomes continuous. We observe how the first O–O coordination peak (at around 2.8 Å) and second peak (at around 4.5 Å) are preserved for most of the pulse duration, up to about 50 fs.

The intermolecular intensity *I*_*inter*_(*q*, *t*) is computed by the Fourier transform of the RDF and shows similar dynamics as the RDF (but not necessarily the same time constants), as seen in Figure 2. The intensity encodes the frequency of the relative distance between O–O coordination peaks. The RDF is truncated at about 10 Å and the computed structure factor therefore captures the local structure of ice. An interesting observation for the higher intensities (5 × 10^18^ and 10^19^) Wcm^–2^, presented in the SI (Figure SI 3) and summarized below, is the buildup of a feature between the two main peaks, driven by the change in interactions and temperature of the system. This is visible at the end of the pulse (45–50 fs) depending on the X-ray intensity, as the two initial strong peaks at *q*_1_ = 1.66 and *q*_2_ = 2.98 Å^–1^ fade out. This effect originates from the real space dynamics in the sample, which enters a new phase without crystalline order.

From the CR simulations using non-local thermodynamic equilibrium approach we estimate the average oxygen ionization and ion temperature as a function of time. After the 50 fs X-ray exposure with the highest intensity of 10^19^ Wcm^–2^, oxygen reaches an average ionization as high as 4 ionizations per atom. The valence electrons (*L*-shell in oxygen), which are involved in molecular bonds, are primarily ionized, as shown in SI. This will cause the oxygen–hydrogen (O–H) bonds to break. We use bond parameters for the O–H bond where the dissociation energy is approximately 4.8 eV (463 kJ/mol).^14^ The ion temperature rises above 4.9 eV (≈57000 K), in figure (SI.14b), for the same intensity within 50 fs of the exposure, reached with a rate of ≈1.14 × 10^18^ K/s. This is an energy comparable to what is needed to break the bonds for a neutral configuration of our system. Since the CR simulations reach temperatures comparable to the binding energies of the molecules, it makes utilizing this model appropriate. The CR model in combination with MD was shown to provide a good agreement compared to experimental scattered signal from nonthermal heating of liquid water.^6^

**Figure 3:**
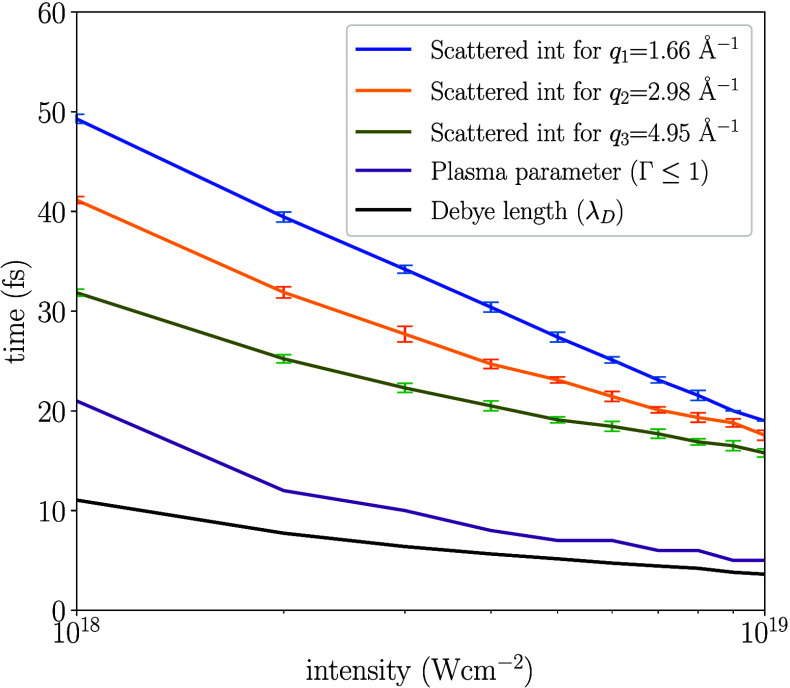
Dynamics of the ice melting passing through the transient crystalline plasma phases, as a function of the incident XFEL intensity. From bottom-up, the lines show: (i) Time-point for the convergence of the Debye length λ_*D*_ that governs the shielding of the Coulomb interactions. (ii) Plasma parameter (Γ = 1) where potential and kinetic energy are equal, below the line the kinetic energy dominates (Γ < 1). (iii) Lines where the scattered intensity at different *q* values drops to *I*(*t*_threshold_) = *I*(*t* = 0)/2. This time-point is averaged over multiple trajectories with different charge distributions, where the error bars correspond to one standard deviation. The plot indicates how the system transitions out from the strongly coupled (condensed matter) phase to a state where the kinetic and potential energy are similar. The results show that the region between the convergence of the plasma parameter and the threshold time of the drop of the scattered intensity for different *q* values correspond to the crystalline plasma states. The point in time above the black line corresponds to an unordered plasma phase.

The state of the plasma is described by the coupling parameter Γ defined as Γ = ⟨*E*_potential_⟩/⟨*E*_kinetic_⟩ and it encodes the coupling between the ions by relating the average electrostatic potential and kinetic energy. The sample starts as a solid which is strongly correlated, however in our plasma model the Coulomb potential energy is low and starts to increase as the ionization increases (Γ < 1). During the simulations these two energies become approximately equal within 20 fs for all intensities studied, as seen in Figure 3. In the same figure, we show the Debye length which encodes the length scales where the Coulomb interaction is reduced. At the initial time-step when the system is a crystal, this quantity is larger than the size of the system. As more free electrons are created and a plasma is formed, the Debye length shrinks to near atomic distances and converges when the value becomes constant (black line). This occurs within the first 10 fs, before any significant atomic movement has occurred. The induced shielding reduces the Coulomb interaction, which leads to the conservation of the crystalline structure. Compared to a simulation where the shielding is turned off, the crystalline structure is preserved for a longer time. In the SI, we discuss the effects and validity of using Debye screening for modeling the Coulomb interaction between the ions for the plasma phase induced in our system. We also demonstrate how the Coulomb force is reduced due to screening at two distances corresponding to the first and second peak in O–O RDF. We find that the Coulomb force an oxygen experiences is reduced with time, which slows down its acceleration, compared to an nonscreened interaction. This is one of the important effects for the success of SFX, since it makes it possible to use longer pulses and still outrun atomic displacement due to radiation damage.^12^

In an X-ray diffraction experiment, the crystalline structure would manifest itself through the Bragg peaks. Peaks at larger scattering angles correspond to shorter distances in the crystal. Dominant peaks at three different scattering angles are visible for *q* values of 1.66 Å^–1^, 2.98 Å^–1^ and 4.95 Å^–1^ as seen in Figure 2. By determining when the signal at different scattering angles disappear, we can estimate the time scale that the crystalline structure is observable. If we further compare to the time point when we expect the system to reach a plasma state, we obtain a time window where the crystalline plasma-ice exists. In Figure 3, we compute the time when the scattered intensity drops below half its value at *t* = 0, for all three *q* values, using intensities between 10^18^ and 10^19^ Wcm^–2^. The figure illustrates that this exotic state of ice persists for up to 30 fs for the highest intensity, and longer time for a less intense pulse. These time scales should be experimentally observable with an X-ray pump–probe setup, or by varying the pulse length and intensity, similar to previous experiments.^6^

**Figure 4:**
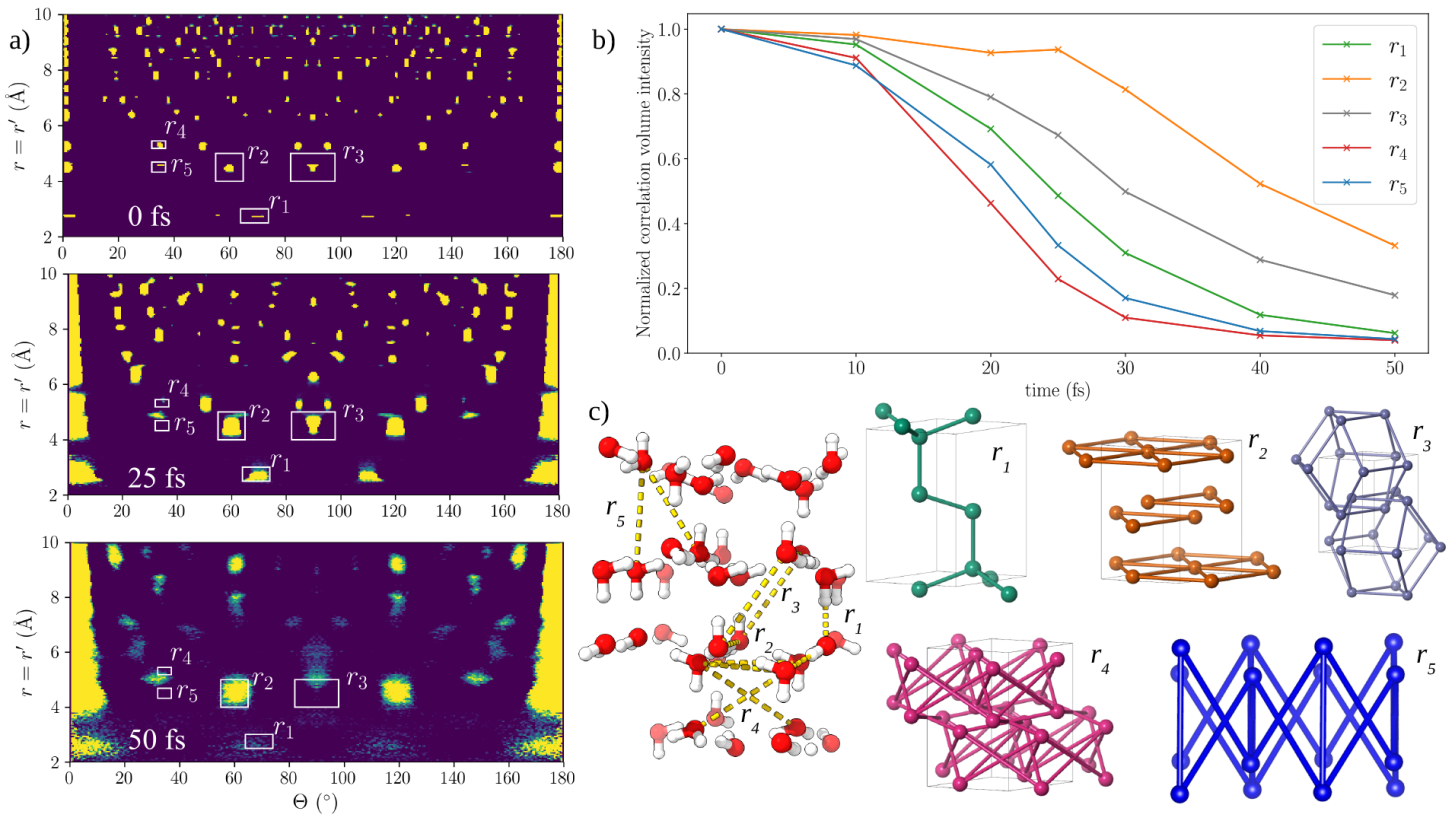
(a) Slice of the pair angular distribution function (PADFs) for *r* = *r*′ of simulated ice structures with intensity 1 × 10^18^ W cm^–2^ at 0, 25, and 50 fs. Areas of high intensity (yellow) indicate frequently observed angular arrangements in the structure. Prominent nearest- and next-nearest neighbor arrangements are labeled *r*_1–5_. (b) Changes in correlation intensity for the peaks *r*_1–5_ indicate the persistence of arrangements related to the hexagonal oxygen packing (all peaks are normalized to 1 at *t* = 0). (c) Geometries of the structure corresponding to arrangements of oxygen atoms (red spheres) and interatomic vectors contributing to each prominent arrangement. The same trend is seen for higher intensities as shown in the SI.

To study whether the melting of the crystalline structure is isotropic, we use the pair-angular distribution function (PADF)^15−17^ as described in the method section. This quantity provides the frequency of 3- and 4-body terms that occur in the structure, distinguished by particular distances and angles. Experimental studies have indicated that structures can respond in a nonisotropic way upon absorption of light and it is therefore interesting to see if we observe nonisotropic processes in our simulations.^18^Figure 4 shows *r* = *r*′ slices for three snapshots for which PADF volumes were calculated. Note that the PADF is a 3D landscape where the distances *r* and *r*′ can be different, here we focus on the *r* = *r*′ plane. These slices show the frequency (yellow corresponding to high frequency) of all arrangements where the lengths of the vectors are identical, making it relatively straightforward to interpret as the frequency of isosceles triangles in the sample (*r*_1–3,5_ are three-point correlations). Starting with the ideal structure at 0 fs in Figure 4, we note that with increasing correlation distance *r*, the slice can be split into nearest and next-nearest neighbor regions. Nearest neighbors have sharply defined distances and angles corresponding to the classic hydrogen-bonded network in hexagonal ice, with *r* ≈ 2.76 Å and Θ ≈ 109°, which we label *r*_1_ (Figure 4c). Increasing *r* further brings us to the next-nearest neighbors in the range 4 < *r* < 6 Å. Three strong contributions can be seen here. The first *r*_2_ at Θ = 120° corresponds to the hexagonal arrangements of oxygen atoms that make up the basal plane of the unit cell. The second, *r*_3_, at Θ = 90°, corresponds to the square arrangements of interatomic vectors between oxygen atoms at (*x*, *y*, *z*) and (x̅, y̅, *z* + 1/2). The next-nearest arrangement with angular signal at (*r*_4_ ≈ 5.25 Å), arises from the four-body arrangements between oxygen atoms stacked along the *c* axis at (*x*, *y*, *z*) and (*x*, *y*, z̅+1/2). The final peak studied is the three-body triangular arrangement at *r*_5_. The angular information encoded in the PADF simulations gives us a unique insight into the directionality of the melting process. Visually, specific contacts in the PADF slices lose intensity more rapidly than others, quantified in Figure 4. Here we show the normalized changes in correlation intensity in volumes of the PADF corresponding to contacts *r*_1–5_. Angular structure within the hexagonal planes (*r*_2_) appears to persist longer than angular structure between hexagonal planes (e.g., *r*_4_). The reason for the stability could be because this 3-body term exists dominantly in one hexagonal plane, compared to the other PADF contacts which could occupy multiple planes (see Figure 1d). There are also some of the *r*_2_ structures where the direction of the hydrogens bonds are exactly the same, shown in figure (SI.13). If all the water molecules involved in the contact interact in a similar way with their connecting hydrogen atoms, this will result in reproducible motion and a longer conservation of the PADF signal. The nearest neighbor correlations (*r*_1_) that average both in-plane and out-of-plane contributions decay fast, which is consistent with scattering experiments where dynamics observed at the largest momentum transfers *q* (smallest real-space distances) decay most rapidly.^19,20^ In agreement with the RDF results, we see the most significant loss of angular structure between 40 and 50 fs, where all initial correlations are lost, except for *r*_2_ and *r*_3_ that have significantly decayed. This is also notable in the PADF for the higher intensities shown in figure (SI.12), and for these intensities the angular correlations are lost more rapidly. We note the nearest-neighbor distance *r*_1_ decays faster compared to the peaks *r*_2_ and *r*_3_, showing that local order on short length scales is lost more rapidly. The *r*_4_ four-body contact also encodes a nearest-neighbor distance and shows an even more rapid decay, slightly faster than the three-body contact at *r*_5_. Several of the studied peaks seem to relocate in a collective manner, introducing new peaks as time evolves. Most notably is the peak which emerges close to *r*_4_ and *r*_5_. This could indicate a new structural configuration where the spherically symmetric potential energy surface has reached a minima. The PADF in Figure 4 corresponds to the lowest computed intensity of 10^18^ Wcm^–2^. By comparing to the results for the higher intensities of 5 × 10^18^ and 10^19^ Wcm^–2^, we note that the relationship between the studied peaks are invariant to the intensity in this range, but the loss of correlation is faster.

## Conclusion

This theoretical study shows that by probing hexagonal ice with an XFEL, the structure can be driven into a state where the crystal to some extent preserves its translational symmetry, while simultaneously entering a plasma phase with charged ions and free electrons. In addition, we have presented results that indicate the ultrafast nonthermal phase transition occurs anisotropically, meaning distinct directions melt at different femtosecond time scales.

In our model, the ultrafast melting dynamics depends on the charge state of the atoms and the size of the Debye sphere that shields the interaction between ions. It is clear the structure is more stable for the lower intensities studied here, since the average charge and the Debye length increase with higher intensities. The charge grows due to a larger number of photons interacting with the sample, as well as Debye length, because of a higher electronic temperature induced by the increase in photoelectrons.

Based on the pair-angle correlations in the oxygen positions we describe anisotropic melting of the crystal, given a model that only contains spherically symmetric interactions, and the assumption that the system rapidly turns into a plasma, breaking the majority of the bonds. We suggest the reason for the anisotropic melting is the geometry of the system and the underlying ion–ion interactions during the nonthermal melting on femtosecond time scales. The interaction between ions changes the structure before the electrons have time to transfer their energy to the ions and thermalize (on picosecond time scales). This effect has been observed experimentally with an IR laser in nonthermal melting of InSb.^21^ The threshold for achieving melting in X-ray crystallography is given by the intrinsic energy scale required to break bonds.^22^ One could therefore predict the melting of an ordered system based on only knowing the geometry of the crystal.

We also see that new correlations appear in the pair-angle distribution in the real space, indicating that a transient structural order can be formed before the sample evaporates. The PADF of the atoms in the real space can in principle be retrieved directly from scattering experiments through angular correlations of the single-shot diffraction patterns^23^ (as suggested in Figure 1) and would provide evidence of the anisotropic melting of crystalline samples or emerging new structures.^17^ Recent experiments have shown that it is feasible to extract correlations from X-ray diffraction experiments as PADF maps.^16^ We envision that transient states of matter could be investigated with this method using an X-ray pump, X-ray probe scheme, at the XFEL facilities around the globe, such as LCLS and EuXFEL.

## Methods

We have used a similar computational approach to what we laid out in our earlier studies.^6,24^ It is a two step model including a first step using collisional-radiative (CR) plasma simulations to describe the time evolution of the ion and electron temperatures through a two-temperature model and ionization in the sample. In a second step we use molecular dynamics (MD) simulations to model structural changes, by providing the time-resolved charge states and free electron temperature/density as input. This hybrid CR/MD approach, called MolDStruct, has been able to reproduce experimental findings.^6,12,25^ To relate our simulations to relevant parameters in coherent diffraction imaging with XFELs, we use 8 keV photon energy, pulse duration of 50 fs with a flat temporal pulse profile, and intensities of range 10^18^ to 10^19^ Wcm^–2^. We simulate the interaction of the photon beam with the ice using a CR code Cretin,^26^ which has in earlier studies proven to be able to simulate these processes with a good agreement to experimental data, specifically in the case of XFEL experiments.^27^ An example input file for CR simulations are available,^6^ and we also provide a web tool for users to explore beam parameters and samples outside the set used in this study.^28^

For the MD we use as in earlier studies^6,24^ the software package Gromacs version 3.3.^29,30^ At each time step in the MD simulation, we iterate through each atom *i* and assign a charge *q*_*i*_ based on the CR data. For each new simulation, we distribute the charge states differently based on a random seed. This enables us to study how different charge distributions in space affect our results. The effect of the free electrons on the Coulomb interaction between the ions is modeled by using the screened form,
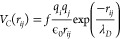
1where ϵ_0_ is the vacuum permittivity, *q*_*i*,*j*_ are the charges, *f* a constant and λ_*D*_ the Debye length. The time-resolved thermalized electron temperature *T*_*e*_ and density *n*_*e*_ are used to calculate the corresponding Debye length, defined as
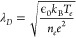
2where *e* is the elementary charge and *k*_*B*_ the Boltzmann constant. We also screened the nonbonded Lennard-Jones (LJ) potential according to the Debye length since it originates from the electromagnetic force
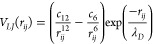
3

For bonded atoms in the equilibrium structure we used a Morse potential^31^ parametrized for the water molecule. Since we model water molecules in a plasma, many-body interactions such as angles and dihedrals are omitted. The parameters for the LJ and Morse potentials were taken from the TIP3P CHARMM model,^14^ and we dynamically changed the parameters for the LJ interaction due to ionization. In this force field, the hydrogen atom is given a radius, making it possible to interact through the LJ potential. When an atom was ionized, the LJ parameters *c*_12_ and *c*_6_ were adapted to take into account the reduction of the ionic radius. This reduction was parametrized using density functional theory calculations in the electronic structure code RSPt.^32^ The hybrid CR/MD simulations were computed with periodic boundary conditions in all dimensions, to simulate an infinite crystal. Nonbonded cutoffs were used to reduce the number of computations. The cutoff radius *r*_*c*_ was set to be the value where the screened Coulomb force was zero. This was calculated based on the Debye length time-averaged over the entire simulation. The crystal was created algorithmically using GenIce,^33,34^ which resulted in a structure with dimensions *a* = 23.469 Å, *b* = 29.414 Å, *c* = 36.146 Å and α = β = γ = 90°. The system included 768 water molecules (2304 atoms).

From the MD simulations we are able to extract the radial distribution functions (RDF)s, which quantifies the probability at particular distances to encounter an atom with respect to a reference particle. It enables the observation of spherically symmetric changes in the structure. Since the MD simulations keep track of the positions and the ionization of all atoms at each time step we can further calculate the diffracted X-ray signal, which can be directly compared to an experiment.^35^

The coherently scattered intensity of a sample from X-rays depends on the partial structure factor between atomic species α and β, *S*_α,β_(*q*, *t*) and form factors *f*_α_(*q*, *t*), *f*_β_(*q*, *t*) as a sum of a self-scattering and intermolecular term
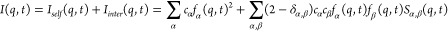
4where the momentum transfer  is related to the real space distance *d* by *d* = 2π/*q*.^36,37^ The parameter α represents the number of different elements present in the sample, with *c*_α_ being the number density of element α. The atomic form factor *f*_α_(*q*) is defined as the Fourier transform of the electron density of an atom, which depends on the electronic configuration. This configuration will change due to the photon-matter interaction. The form-factor *f*_α_(*q*, *t*) was computed by weighting all observed electronic configurations given by the CR simulations,
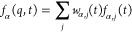
5where *w*_α,*j*_ = [0, 1] (*∑*_*j*_*w*_α,*j*_ = 1) is the weight and *f*_α,*j*_ the form-factor of element α and the electronic configuration *j*. The electron density used to compute the *f*_α,*j*_ was derived using wave functions in *RSPt*.^32^ The partial structure factors *S*_α,β_ can be calculated as the Fourier transform of the time dependent RDF *g*_*αβ*_(*r*, *t*)^37^ as
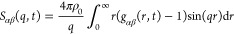
6

The scattered intensity calculated by eq 4 only provides 1D information in reciprocal space. We obtained angular details from diffraction patterns of single crystals using a newly developed software package that includes radiation damage from atomic displacement and ionization. Trajectories from MD simulations provide the motion, while time-dependent form-factors *f*_α,*j*_(*q*, *t*) encode electronic changes. The simulated diffraction patterns with no radiation damage (simulation time-step 0) were validated against the X-ray simulation tool Condor.^38^ The pulse parameters for this analysis followed those used in CR simulations. We placed a virtual 1024,1024-pixel array detector with 200 μm pixel size at a distance of 70 mm. The angular correlations of the simulated diffraction patterns were calculated using open-source software *loki*(39) by interpolating the pixel intensities to polar coordinates in steps of 0.01 Å^–1^ and 1° and computing the intensity correlation ⟨*I*(*q*,ϕ)*I*(*q*,ϕ + Δ)⟩_ϕ_ averaged over all angles ϕ.

To detect if there is any angular dependence in the melting process, we used pair angle distance functions (PADF) .^15,17^ Trajectories from 0, 10, 20, 25, 30, 40, and 50 fs were used to generate ±1 supercells containing only oxygen atoms to give a real-space probe distance of 10 Å corresponding to the limit of the RDFs used shown in Figure 2 in the article. For all the atoms in the subject cell we calculate all interatomic vectors and iterate through this set of interatomic vectors, calculating the angles between each pair and populating the PADF histogram (seen in manuscript Figure 4) with these values. Since all the atoms in the sample are the same type (O), the correlation intensity does not require scaling by the atomic form factor. In practice we do not calculate the correlations for all interatomic vectors but instead iterate until the cosine similarity of the *n* and *n* – 1 PADF volumes falls below a cutoff <10^–11^, which was determined from a series of convergence tests using one trajectory. The normalized intensity plots in the manuscript Figure 4 are computed from an initial box which encapsulates the peak at the initial time-step, where the result one gets depends on the volume integration of this square. We therefore computed the volume integration systematically by tracking the peak as the system starts moving. We define the size of the new region that is integrated as the coordinates (*r* = *r*′ and θ) which are above a defined tolerance times the maximum peak at the current time-step.

## Supporting Information Available

Information on water structure, additional methods on atomic model convergence, isotropic intensity calculations, coherent diffraction calculations, and molecular dynamics interactions, additional results for scattering intensity, charge dynamics and pair angle distribution function, and additional discussion points related to the validity of choices made for modeling the ice structure (PDF)
