# Supplementary material for: Theoretical Studies of Anisotropic Melting of Ice Induced by Ultrafast Nonthermal Heating
Source: ACS Phys Chem Au. 2024 May 8;4(4):385–92. doi: 10.1021/acsphyschemau.3c00072 (PMC11274275; doi:10.1021/acsphyschemau.3c00072)
Supplement: Supplementary file 1 — pg3c00072_si_001.pdf [file pg3c00072_si_001.pdf]

Theoretical studies of anisotropic melting of ice induced by ultrafast non-thermal heating

Ibrahim Dawod,[*,†,‡,@] Kajwal Patra,[†,@] Sebastian Cardoch,[†] H. Olof Jönsson,[¶] Jonas A. Sellberg,[¶] Andrew V. Martin,[§] Jack Binns,[§] Oscar Grånäs,[†] Adrian P. Mancuso,[‡,∥,⊥] Carl Caleman,[†,#] and Nicusor Timneanu[*,†]

†*Department of Physics and Astronomy, Uppsala University, Box 516, SE-751 20 Uppsala, Sweden*

‡*European XFEL, Holzkoppel 4, DE-22869 Schenefeld, Germany*

¶*Department of Applied physics, KTH Royal Institute of Technology, SE-106 91, Stockholm, Sweden*

§*School of Science, RMIT University, Melbourne, Victoria 3000, Australia*

∥*Diamond Light Source, Harwell Science and Innovation Campus, Didcot OX11 0DE, UK*

⊥*Department of Chemistry and Physics, La Trobe Institute for Molecular Science, La Trobe University, Melbourne, Victoria 3086, Australia*

#*Center for Free-Electron Laser Science, Deutsches Elektronen-Synchrotron, Notkestraße 85 DE-22607 Hamburg, Germany*

@*These authors have contributed equally to the work.*

E-mail: ibrahim.dawod@physics.uu.se; nicusor.timneanu@physics.uu.se

Additional information

The structure of ice

The length scales which describe the RDF peaks are shown in the table SI. 1.

Table SI. 1: Table of the first three peaks for the radial distribution function of the ice structure, as computed using GenIce[1]. The values are in nanometers.

Peak	O-O	O-H	H-H
r_1	0.276	0.096	0.152
r_2	0.452	0.18	0.232
r_3	0.528	0.322	0.302

Additional methods

Atomic model convergence for CR simulation

We model the interaction between photons and matter based on atomic physics, even though the bound electrons in the water molecule are more accurately described by molecular orbitals. This is motivated by the fact that core electrons are rather localized, especially when the atom is positively charged. Furthermore, the error in the ionization energy and cross-sections from neglecting molecular orbitals will be small since the molecule will be in a plasma, which causes many other perturbations to the orbitals of the molecule.

The screened hydrogenic model (SHM) in Cretin[2] is developed by averaging levels from a more complicated atomic model (which for instance can contain l quantum numbers) where the atom is only described by its n quantum numbers. The resulting model can still be reduced, by for instance reducing the total number of available n levels, limiting the number of core holes in higher n quantum number levels, constraining the total number of electronic excitations and lowering the largest principal quantum number used for excitations.

We studied the convergence of the free electron temperature as a function of the number of principal quantum numbers in the model. The convergence was conducted by simulating the

interaction between the pulse and the sample, using the largest intensity in the manuscript (10^{19} W/cm^2). The average electron temperature of the last 10 fs of the 50 fs pulse was used for analyzing the convergence. The result is shown in figure (SI. 1). The figure shows that the electron temperature has a small change with increasing principal quantum number (PQN). Thus, the final model of oxygen was set to have a PQN of 10. We allowed for 8 electrons to be excited in the atom at the same moment. Furthermore, the model was allowed to contain two-core hole states. The convergence study allowed us to utilize a reduced model, which provided a speed-up in the computation of the production simulations which spanned multiple intensities.

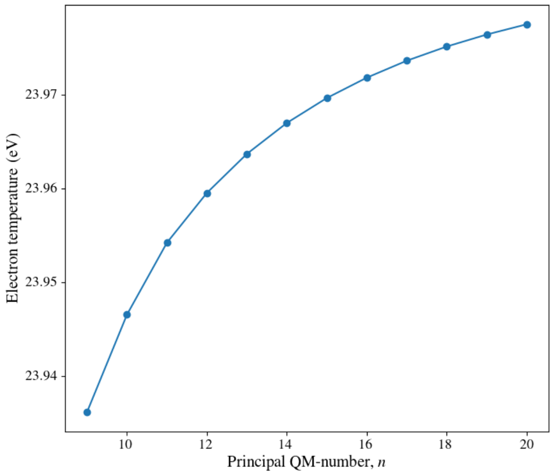

Fig. SI. 1: Electronic temperature as a function of principal quantum number n used in the atomic model of oxygen.

Isotropic scattered intensity

The time-resolved scattered intensity from ice can be calculated by the formula[3]

$$I(q,t) = \sum_{\alpha} c_\alpha f_\alpha(q,t)^2 + \sum_{\alpha,\beta} (2-\delta_{\alpha,\beta}) c_\alpha c_\beta f_\alpha(q,t) f_\beta(q,t) S_{\alpha,\beta}(q,t) \, , \tag{SI. 1}$$

where the first term is the self-scattering term $I_{self}(q,t)$, which does not provide any information regarding the relative position of the atoms. The second part corresponding to the inter-molecular

term $I_{inter}(q,t)$ encodes structural information, through the use of summation of the partial structure factors $S_{\alpha,\beta}$. The quantity $S_{\alpha,\beta}$ is computed using the RDFs $g_{\alpha,\beta}(r)$ in the hybrid plasma/molecular dynamics simulations

$$S_{\alpha\beta}(q) = \frac{4\pi\rho_0}{q} \int_0^\infty r(g_{\alpha\beta}(r) - 1)\sin(qr)\mathrm{d}r \; . \tag{SI. 2}$$

Given that the two atomic species we have in our system is oxygen (O) and hydrogen (H), we get $\alpha, \beta = O, H$. Therefore, we can write the structure factor due to the RDF of O-H being symmetric $g_{OH}(r) = g_{HO}(r)$ as,

$$I_{inter}(q,t) = c_O^2 f_O(q)^2 S_{OO} + c_H^2 f_H(q)^2 S_{HH} + 2c_O c_H f_O(q) f_H(q) S_{OH} \; . \tag{SI. 3}$$

Coherent diffraction

We computed the coherent diffraction from single crystals using the following equation

$$I(\boldsymbol{q},t) = I_0(t)\,\Omega(\boldsymbol{q})\,|F(\boldsymbol{q},t)|^2\,(d\sigma/d\Omega)_T \;, \tag{SI. 4}$$

where $I_0(t)$ is the illumination fluence assumed to be homogeneous across the sample, $\Omega(\boldsymbol{q})$ is pixel-wise detector solid angle, $F(\boldsymbol{q},t)$ is the time-dependent structure factor, and $(d\sigma/d\Omega)_T = r_e^2\,|\hat{\varepsilon}\cdot\hat{\varepsilon}'|^2$ is the Thompson scattering cross-section with $\hat{\varepsilon}$ and $\hat{\varepsilon}'$ representing the polarization of the incident and scattered X-rays, respectively[4]. The structure factor is defined as

$$F(\boldsymbol{q},t) = \sum_i c_i(t)\, f_{0,i}(q)\, e^{i\boldsymbol{q}\cdot\boldsymbol{r}_j(t)} \; . \tag{SI. 5}$$

Here $c_i(t)$ represent the population and $f_{0,i}(q)$ the atomic scattering function of the electronic configuration i for example O–$1s^1 2s^2 2p^4$. Similar methods to determine structure factors have been

reported in the literature[5]. We assumed spherical symmetry on the atomic scattering function

$$f_{0,i}(q) = 4\pi \int dr\, |\psi_i(r)|^2 \,\mathrm{sinc}(qr)\, r^2 \,. \qquad \text{(SI. 6)}$$

We computed the radial atomic wavefunctions $\psi_i(r)$ for each configuration with electronic structure code RSPt and their time-dependent populations with collisional radiative code cretin. The diffraction simulations ran in Julia 1.7.2 taking advantage of the non-uniform fast Fourier transforms package FINUFFT 3.1.0[6], which allowed us to sample the real space at atomic positions. We sliced the incident beams with 10 different energies within 1% of the beam's photon energy to represent the bandwidth and averaged the resulting diffraction patterns incoherently. We disregarded polarization, quantum efficiency effects, and discretization of the photons upon detection. We used a tolerance of 1×10^{-10} to solve the fast Fourier transform.

Molecular dynamics bonded interactions

For running classical molecular dynamics simulations, a force field, which describes the interactions between the atoms in the system, is needed. In our simulations we assume that the sample is a plasma, and therefore we must alter the interaction potentials used. Since the assumption of Cretin is that the sample is plasma, the method is only valid if the native sample transitions into the plasma phase in a time-scale which is short compared to the time-scale of atomic motion. We note that during the first 1-2 fs of the interaction between the photons and the matter the sample is not described by a plasma, but is so after, as can be observed in figure (SI. 11).

Since the atoms occupy essentially their native positions in the time it takes to reach the plasma phase, we are sure that we can base our physical description of the system on plasma physics. Even though a plasma usually describes a system where bonds do not exist, and the probe will turn the sample into a plasma, the charge distribution will depend on the parameters used, and thus it is likely that there will be a fraction of atoms which are neutral, as is proven for both hydrogen and oxygen in figures (SI. 9) and (SI. 10). In this case, one still wants the bonds to be intact.

Therefore, we decided to use Morse potentials[7] for atoms which are bonded in the native system. The potential errors introduced by the parameters used are essentially removed by starting the simulations at 0 K. This results in that the propagation of the molecular dynamics is dictated solely by the collisional-radiative calculations. The use of the Morse potential is further motivated by the fact that one needs to make sure that the atoms' electronic clouds repel as the atoms approach each other. The Morse potential is defined as

$$V_{Morse}(r,t) = D_e\left(1 - \exp(-\beta(r - r_e)\right)^2 \tag{SI. 7}$$

where the depth of the well is D_e, the well steepness β and the equilibrium distance r_e all depend on the two atoms involved. We validated the parameters by checking the stability of a simulation starting at 0 K using only the Morse and Lennard-Jones potential. We observed that the temperature did not increase and therefore concluded that the force field would be adequate for running the plasma simulations. The converged parameters were then used in the production simulations for running the hybrid plasma/MD simulations of ice.

The screened non-bonded interactions

In this section, we present the potentials that are used to compute the non-bonded interactions between the atoms. The screened Coulomb potential was defined as[8]

$$V_C(r,t) = f\frac{q_1(t)q_2(t)}{r(t)}\exp(-r(t)/\lambda_D(t)) \tag{SI. 8}$$

where $f = 138.935458$ kJ mol^{-1}nm e^{-2}, and the scalar force was given by $F = -\nabla V(r)$

$$F_C(r,t) = f\frac{q_1(t)q_2(t)(\lambda_D(t) + r(t))}{\lambda_D(t)r(t)^2}\exp(-r(t)/\lambda_D(t)). \tag{SI. 9}$$

The screened Lennard Jones potential is defined as

$$V_{LJ}(r,t) = \left(\frac{c_{12}}{r(t)^{12}} - \frac{c_6}{r(t)^6}\right)\exp(-r(t)/\lambda_D(t)) \tag{SI. 10}$$

and the force

$$F_{LJ}(r,t) = \exp(-r(t)/\lambda_D(t))\left(c_{12}\left(\frac{1}{\lambda_D(t)r(t)^{13}} + \frac{13}{r(t)^{14}}\right) - c_6\left(\frac{1}{\lambda_D(t)r(t)^7} + \frac{7}{r(t)^8}\right)\right). \tag{SI. 11}$$

The parameters c_6 and c_{12} depend on the two atom types involved. In our simulations, they dynamically change due to the reduction of bound electrons in the atoms. As one removes electrons from the atoms, the ionic radius will reduce and the atoms can reach closer to each other. Therefore, the parameters are changed such that this effect is present. Furthermore, since the Lennard-Jones interaction is an interaction originating from the electromagnetic force, we apply the same screening factor like in the Coulomb potential case, in order to get the effects of the electronic environment.

Given the important length scales as described by the peaks in the RDFs, we compute the screened force at the first two peaks ($r_x = r_1, r_2$) as a function of time, normalized by the regular Coulomb interaction,

$$F_{norm}(r_x,t) = \frac{(\lambda_D(t) + r_x(t))}{\lambda_D(t)}\exp(-r_x(t)/\lambda_D(t)) \tag{SI. 12}$$

Figure (SI. 2) shows how the forces at these length scales are greatly reduced due to the screening of the free electrons, as computed by equation (SI. 12). Leaving out the effect of the electron cloud would result in an increased Coulomb force and thus a much more rapid expansion of the system.

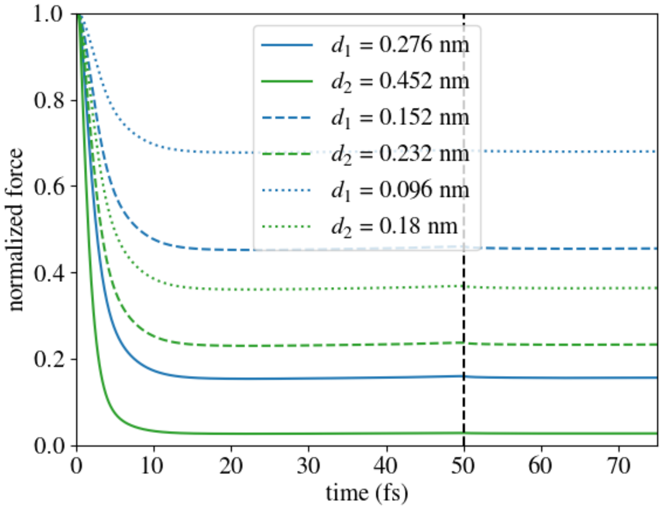

Fig. SI. 2: Screened Coulomb interaction normalized by the regular Coulomb force at different length scales. The data for the Debye length is from a simulation with an intensity of 1e18 Wcm^{-2}. The filled line is for O-O, dashed is for H-H and dotted for O-H.

Additional results

The time-resolved RDF used in the computation of the partial structure factor for O-O that are not present in the manuscript is shown in figure SI. 3. The inter-molecular part of the intensity computer with equation (SI. 1) is show in figure SI. 4 for oxygen-oxygen bonds and in figure SI. 5 for oxygen-oxygen, oxygen-hydrogen and hydrogen-hydrogen bonds.

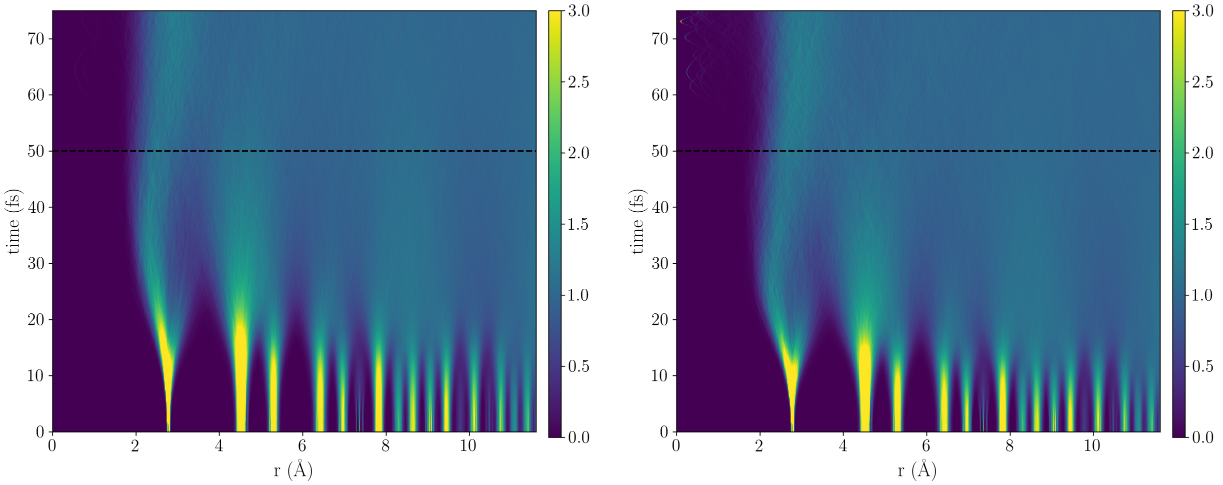

(a) 5e18 W/cm^{-2}.

(b) 1e19 W/cm^{-2} .

Fig. SI. 3: RDFs from the hybrid plasma/MD simulation for O-O.

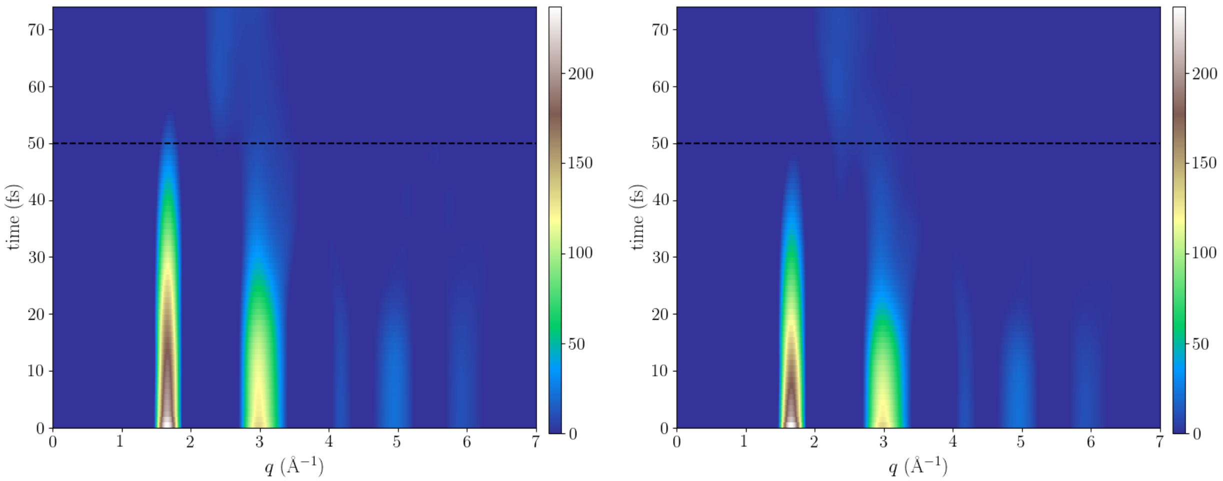

(a) 5×10^{18} Wcm^{-2} .

(b) 10^{19} Wcm^{-2}.

Fig. SI. 4: Intermolecular scattering term $I_{inter}(q,t)$ from the hybrid CR/MD simulation for O-O.

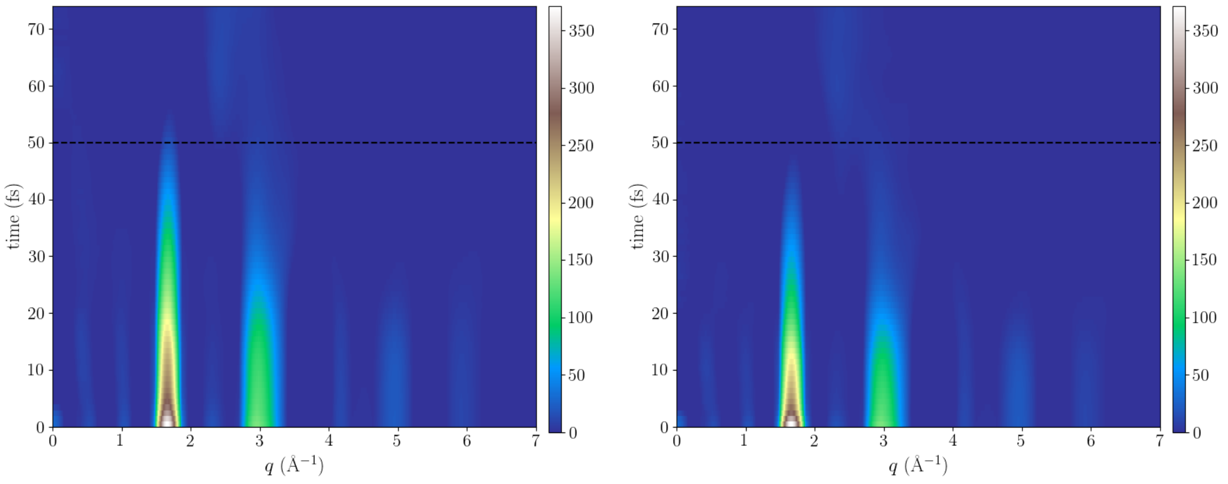

(a) 5×10^{18} Wcm^{-2} .

(b) 10^{19} Wcm^{-2}.

Fig. SI. 5: Inter-molecular scattering term $I_{inter}(q,t)$ from the hybrid CR/MD simulation for O-O, O-H and H-H.

Coherent diffraction

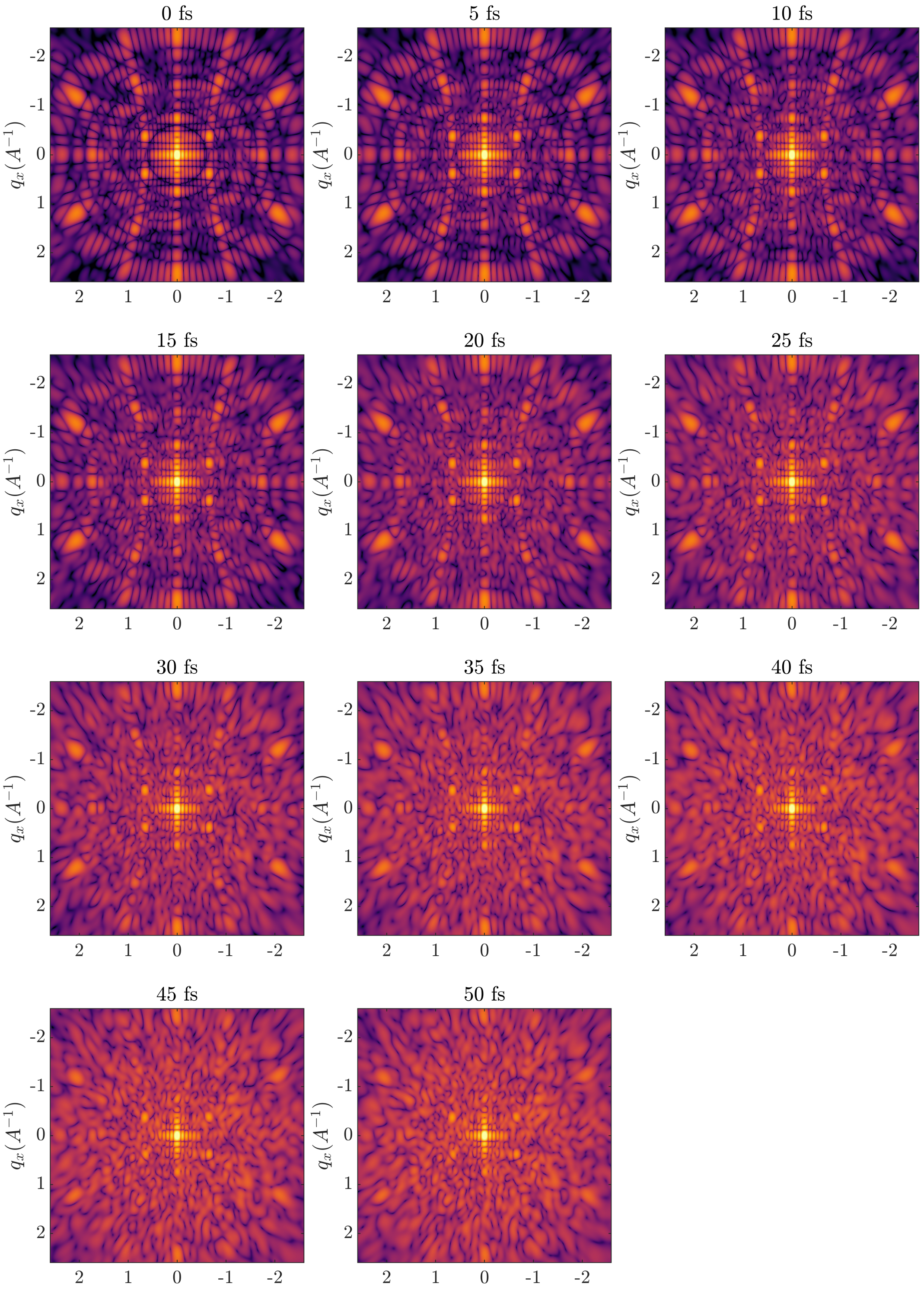

Fig. SI. 6: Snapshots of diffraction patterns for an XFEL intensity of 10^{18} $\mathrm{Wcm^{-2}}$, taken every 5 fs during the 50 fs pulse.

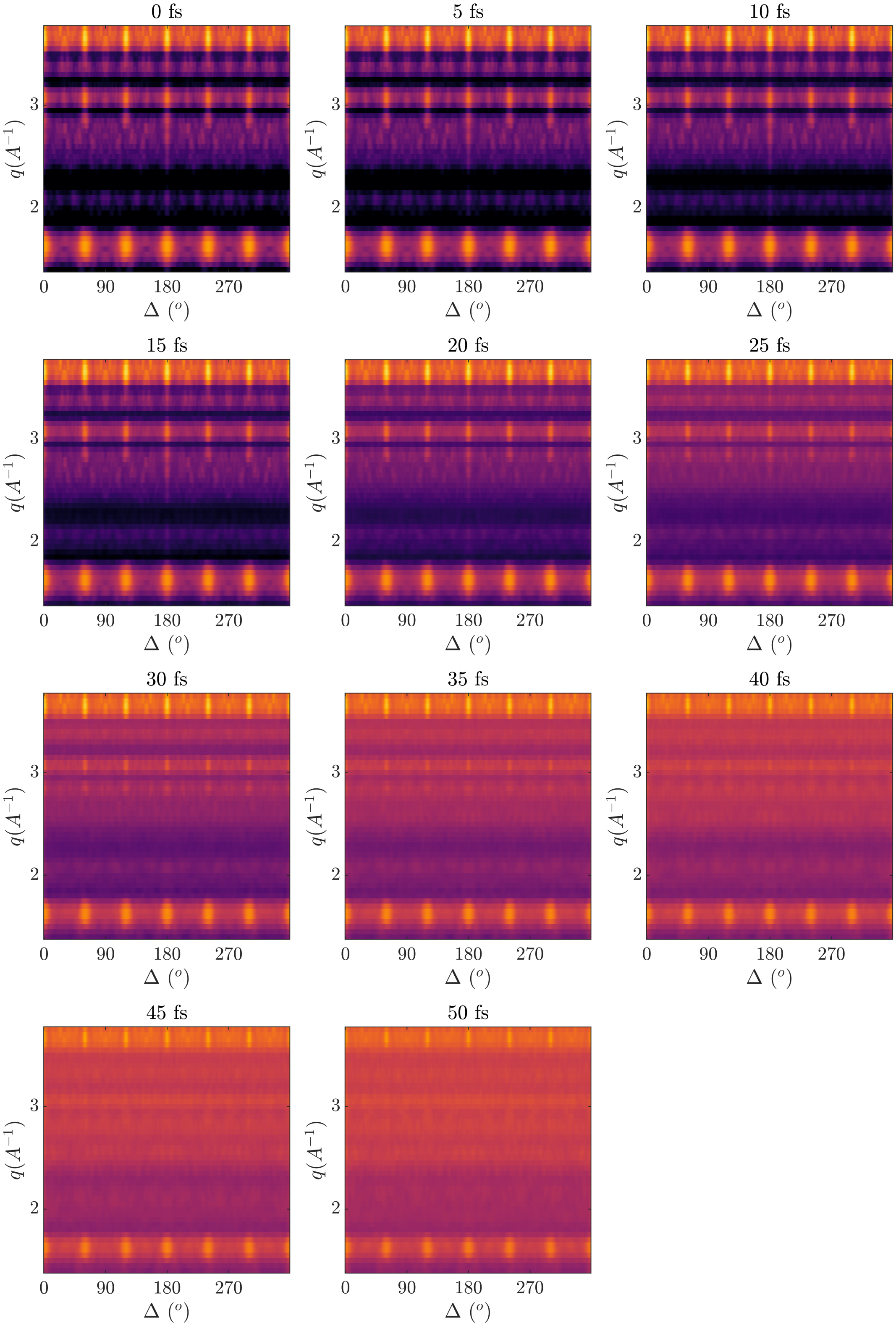

Fig. SI. 7: Angular correlation of the diffraction patterns for an XFEL intensity of 10^{18} $\mathrm{Wcm^{-2}}$.

Charge dynamics in CR simulations

The main data collected from CR simulations to run classical molecular dynamics (MD) in GROMACS[9] is the fractional charge state of each atomic species (as seen in figure (SI. 9), the free electron temperature and density. Blow we show the charge dynamics for oxygen and hydrogen atoms at different conditions.

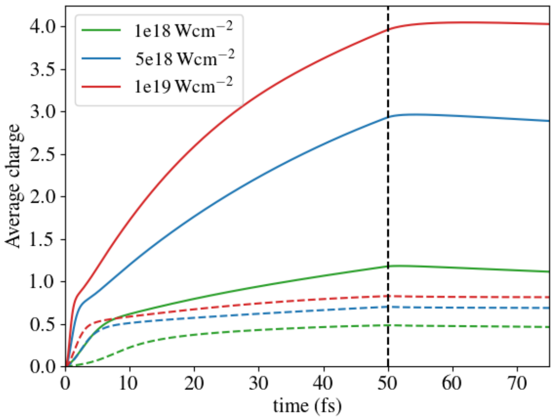

Fig. SI. 8: Average charge of oxygen (filled lines) and hydrogen (dashed lines) for a 50 fs flat shaped pulse XFEL pulse with 8 keV photon energy, shown for different XFEL intensities. The range of intensity is chosen to achieve an average ionization of oxygen that will allow for coherent scattering during the X-ray pulse.

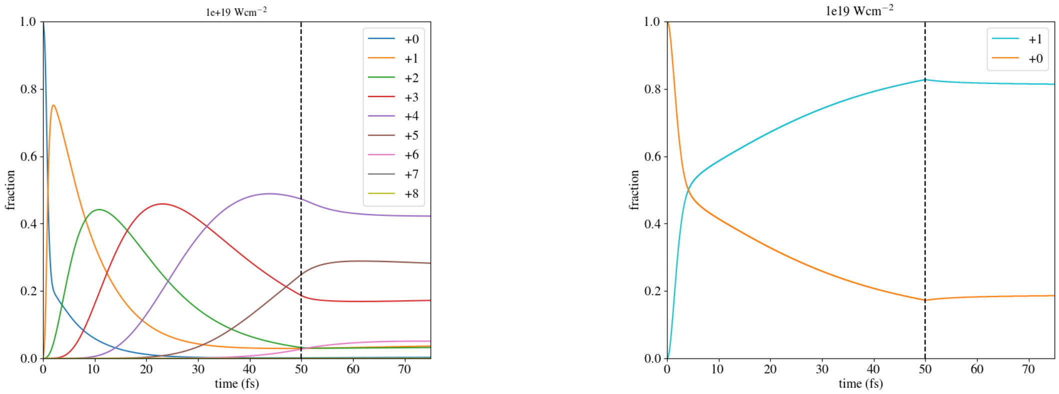

(a) Oxygen.

(b) Hydrogen

Fig. SI. 9: Example of the fractional charge distribution for an intensity of 10^{19} Wcm^{-2} and photon energy 8 keV.

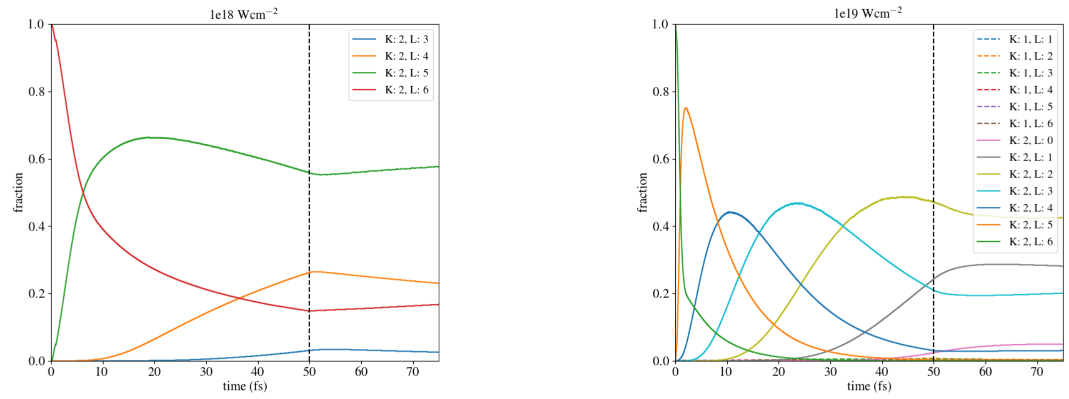

(a) 10^{18} Wcm^{-2}.

(b) 10^{19} Wcm^{-2}.

Fig. SI. 10: Fractional electronic occupation of oxygen, for the lowest (10^{18} Wcm^{-2}) and highest (10^{19} Wcm^{-2}) intensities. The value in the legend corresponds to the number electrons in the K and L electronic shells. It can be observed the fraction of core holes is negligible for these pulse parameters.

Debye length and plasma parameter

The electron density from CR simulations is used to compute the Debye length (shown in figure (SI. 11a), which screens the electromagnetic interactions. The data from Cretin is sampled with the same time-step as used in the MD simulations, which was chosen to be 10^{-17}s.

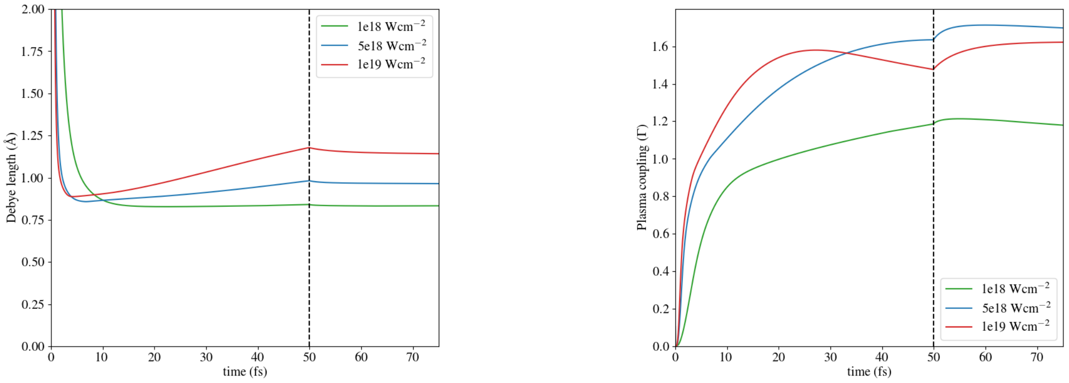

(a) Debye length (λ_D).

(b) Plasma parameter (Γ).

Fig. SI. 11: Debye length and plasma parameter for for an intensity of 10^{18}, 5×10^{18} and 10^{19} W/cm^2 and photon energy 8 keV.

Pair angle distribution function

The figure (SI.SI. 12) shows the pair-angle distribution function (PADF) changes as a function of time for three different intensities. The PADFs geometries of r_{1-5} are described in the main article. Figure (SI.SI. 13) shows in particular the PADF contact r_2 from different angle (top view, hexagonal ice).

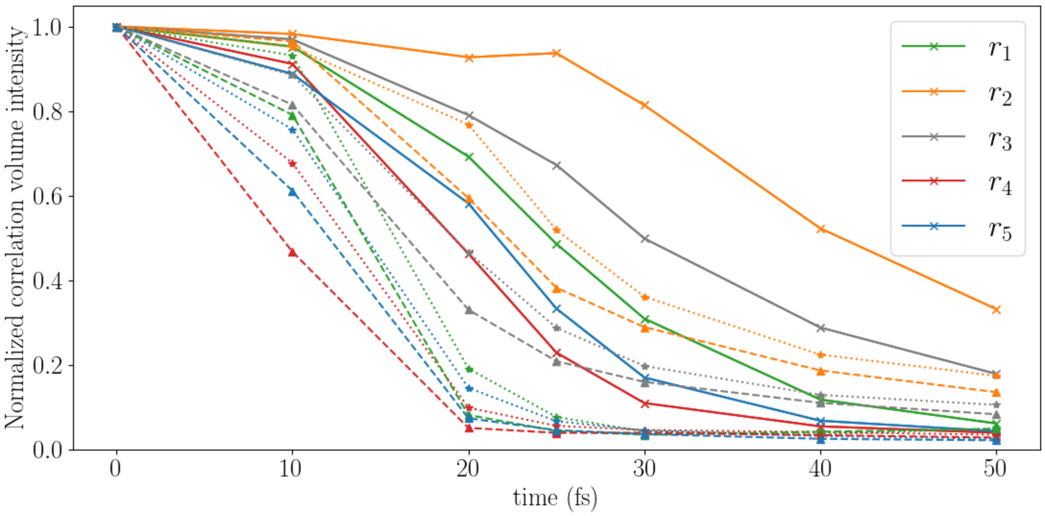

Fig. SI. 12: Comparison of the PADF for three different intensities: 10^{18} Wcm^{-2} (filled lines), 5×10^{18} Wcm^{-2} (dotted lines) and 10^{19} Wcm^{-2} (dashed lines).

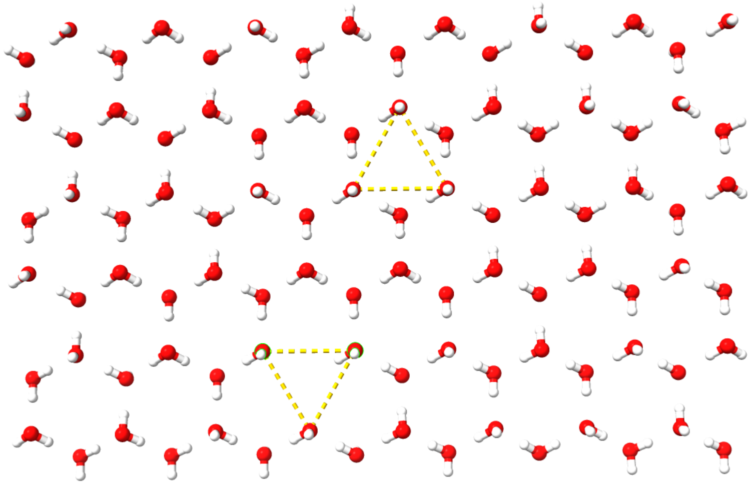

Fig. SI. 13: Slice of one plane of the ice structure showing the geometry of the molecules for the r_2 PADF contact.

Additional discussion

Debye screening to model Coulomb interactions

For the plasma conditions studied here, the Debye screening model is not valid for all length scales. Debye screening is appropriate for length scales much smaller than the dimensions of the system, which is always true for our simulations. However, we may not have enough charged particles in the Debye sphere to make the concept statistically significant as the Debye length becomes shorter. Therefore, the concept of Debye screening breaks down close to the ion. An ion sphere model instead is most appropriate for a strongly coupled condensed matter system closer to the ion. For long range screening where we approach long distance away from the ion, the Debye length will be statistically valid[10]. We believe the way we incorporate the effects of the electrons on the ion-ion Coulomb interaction is a good approximation for this system. The complete mathematical form of the screening is something else than we use[10], but our simplified formula will can capture well the essence of the screening phenomena. Comparing these two screening models in water close to the ion, we do not see enough difference to motivate using a hybrid screening model[10]. Finally, when

simulating the same system with and without screening, the only notable difference in the dynamics is the time-scale for the melting, and not the process of melting.

Stewart-Pyatt continuum lowering

In our collisional-radiative calculations used to compute the photon-matter interactions, we utilize the Stewart-Pyatt model for the continuum lowering.There is some debate in the literature about the appropriateness of the Steward-Pyatt model to capture continuum lowering effects in favor of the Ecker-Kroll model[11,12]. Other papers suggest that Steward-Pyatt is most appropriate or that lowering is well captured by these parametric models[13,14]. Recent developments in the field involve the use of density functional theory or other methods[15,16]. To our knowledge there is no simple model that can be implemented in collisional-radiative simulations.The relevant physics to capture the transition from a degenerate plasma (condensed matter like) to warm dense matter implemented in Cretin[2] include: inclusion of the effects of electron degeneracy on the rates, opacities, emissitives and collisional ionization. This code has previously been employed to study x-ray induced dynamics in matter with comparison to experiments[17] and has along with appropriate atomic data been benchmarked to other similar codes[18]. Similar to our pipeline, other codes[19,20] use MD simulation supplemented by ionization dynamics from rate equations to model x-ray induced atomic damage in crystalline systems. While the validity of the SP model is been debated, it still gives adequate results which can be used to make conclusions in this study. Our observable is not the ionization state (or free electron dynamics), but the diffraction pattern, which depends on electronic occupation and atomic positions. We therefore do not expect much differences compared to a different continuum lowering model.

Temperature and root-mean-square displacement comparison between CR and MD simulations

Information regarding structure is not available in Cretin. The dynamics of the structure are instead studied by taking data from Cretin to run MD in GROMACS. A advantage of using MD is that we can study dynamics for a system which is not isotropic. Thus, one should expect that a quantity such as temperature should be at least on the same order between the two methods. In figure (SI. 14) we compare the temperature from the MD and CR calculations. We note that the temperatures are indeed of similar order of magnitude with time for the highest intensity. Even though temperatures are only of the same order of magnitude, the differences are not expected to change the interpretation of the MD results. Furthermore, we note that the temperature evolution of different trajectories in the MD simulations, where the charge distribution is assigned differently in space, are reproducible.

We started simulating 10 different trajectories with different random seeds. Each simulation distributed the charge states as seen in figure (SI. 9) differently. Out of the 10 trajectories, some of the simulations had to be removed since the temperatures increased in a single time-step in a non-physical way. We are not completely certain of its origin, but we theorize that since electron recombination causes a change in the Lennard-Jones parameters, it may be that two atoms that are at separation d_{t_1} at time t_1, will be stable (i.e. no large LJ-forces) but at time t_2, when one or both atoms have had recombination of an electron, will make this separation for the new LJ-parameters more repulsive and thus result in large forces. This would, in turn, lead to large spikes in the temperature.

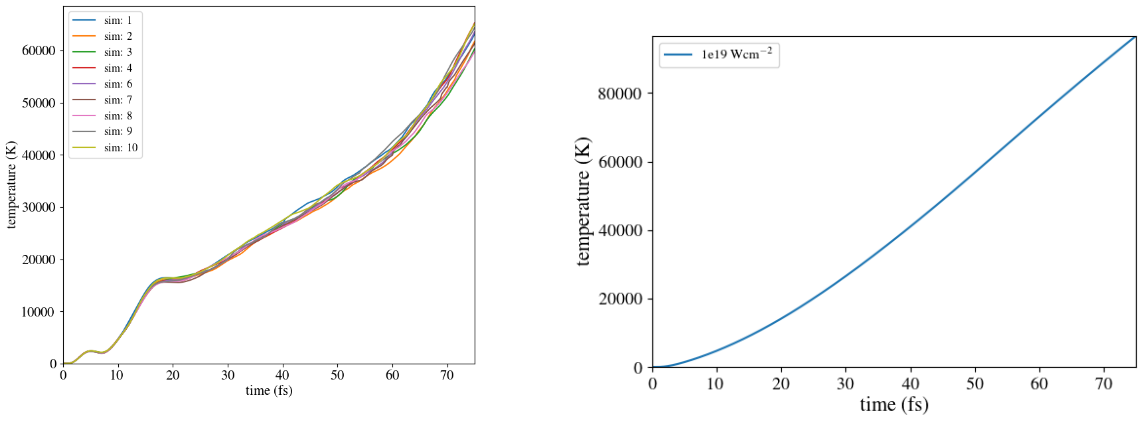

(a) GROMACS. (b) Cretin.

Fig. SI. 14: The ion temperature for an intensity of 1e19 W/cm^2 and photon energy 8 keV.

Bond breaking with *ab-initio* methods

In our previous work, where the dynamics of charged amino-acids and peptides were studied, we concluded that some bonds could be robust to ionization[21,22]. For the water molecule, we justify the use of Morse potentials (bonds) by running density functional theory (DFT) simulations scanning different net charges (+0, +1, +2, +3, +4) and detecting the threshold charge for breaking the bonds. We performed all-electron unrestricted Kohn-Sham calculations in ORCA[23], with B3LYP as the exchange correlation functional and TZVPP as the basis set for both energy minimization and the molecular dynamics calculations. A single trajectory starting at 300 K was computed for 75 fs, where each time-step was 0.5 fs. The results in figure (SI. 15) show that both O-H bonds are intact for up to +2 charge, while for +3, one of the bonds breaks. For higher charges than +3, both bonds break. Thus, we show using *ab-initio* molecular dynamics, that the bonds in water can be intact, even though the molecule is charged.

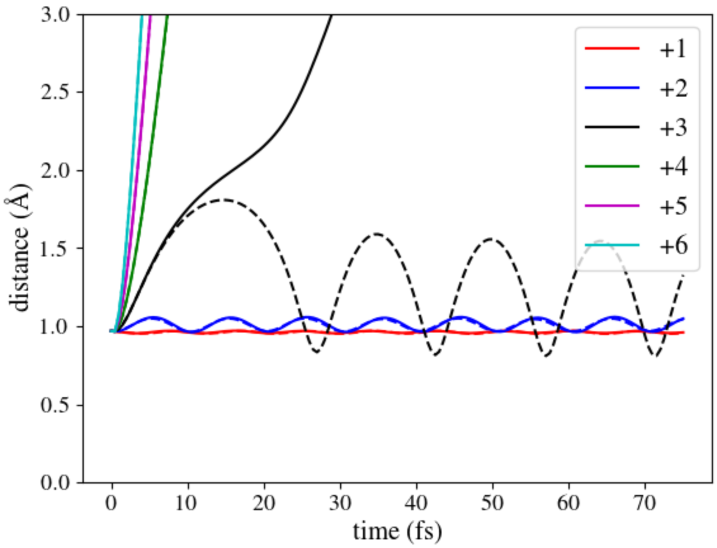

Fig. SI. 15: Distance between O-H as a function of time from *ab-initio* molecular dynamics of charged H_2O, as calculated by ORCA. The dotted/non-dotted line each show one of the bonds to the hydrogens.

References

(1) Matsumoto, M.; Yagasaki, T.; Tanaka, H. GenIce: Hydrogen-Disordered Ice Generator. *Journal of Computational Chemistry* **2018**, *39*, 61–64.

(2) Scott, H. A.; Mayle, R. W. GLF - A simulation code for X-ray lasers. *Applied Physics B* **1994**, *58*, 35–43.

(3) Soper, A. K. Joint structure refinement of x-ray and neutron diffraction data on disordered materials: application to liquid water. *Journal of Physics: Condensed Matter* **2007**, *19*, 335206.

(4) McMorrow, D.; Als-Nielsen, J. *Elements of Modern X-Ray Physics*; John Wiley & Sons, Incorporated: Hoboken, UNITED KINGDOM, 2011.

(5) Ho, P. J.; Daurer, B. J.; Hantke, M. F.; Bielecki, J.; Al Haddad, A.; Bucher, M.; Doumy, G.; Ferguson, K. R.; Flückiger, L.; Gorkhover, T.; et al. The role of transient resonances for ultra-fast imaging of single sucrose nanoclusters. *Nature Communications* **2020**, *11*, 167.

(6) Barnett, A. H.; Magland, J. F.; Klinteberg, L. a. A parallel non-uniform fast Fourier transform library based on an "exponential of semicircle" kernel. 2019; http://arxiv.org/abs/1808.06736, arXiv:1808.06736.

(7) Morse, P. M. Diatomic molecules according to the wave mechanics. II. Vibrational levels. *Phys. Rev.* **1929**, *34*, 57–64.

(8) Chen, F. F. *Introduction to plasma physics and controlled fusion*; Springer, 1984; Vol. 1.

(9) Lindahl, E.; Hess, B.; Van Der Spoel, D. GROMACS 3.0: a package for molecular simulation and trajectory analysis. *Molecular modeling annual* **2001**, *7*, 306–317.

(10) Murillo, M. S.; Weisheit, J. C. Dense plasmas, screened interactions, and atomic ionization. *Physics Reports* **1998**, *302*, 1–65.

(11) Ciricosta, O.; Vinko, S.; Barbrel, B.; Rackstraw, D.; Preston, T.; Burian, T.; Chalupskỳ, J.; Cho, B. I.; Chung, H.-K.; Dakovski, G.; et al. Measurements of continuum lowering in solid-density plasmas created from elements and compounds. *Nature communications* **2016**, *7*, 11713.

(12) Vinko, S.; Ciricosta, O.; Wark, J. Density functional theory calculations of continuum lowering in strongly coupled plasmas. *Nature communications* **2014**, *5*, 3533.

(13) Hoarty, D.; Allan, P.; James, S.; Brown, C.; Hobbs, L.; Hill, M.; Harris, J.; Morton, J.; Brookes, M.; Shepherd, R.; et al. Observations of the effect of ionization-potential depression in hot dense plasma. *Physical review letters* **2013**, *110*, 265003.

(14) Fletcher, L.; Kritcher, A.; Pak, A.; Ma, T.; Döppner, T.; Fortmann, C.; Divol, L.; Jones, O.; Landen, O.; Scott, H.; et al. Observations of continuum depression in warm dense matter with x-ray Thomson scattering. *Physical review letters* **2014**, *112*, 145004.

(15) Son, S.-K.; Thiele, R.; Jurek, Z.; Ziaja, B.; Santra, R.; et al. Quantum-mechanical calculation of ionization-potential lowering in dense plasmas. *Physical Review X* **2014**, *4*, 031004.

(16) Rosmej, F. Ionization potential depression in an atomic-solid-plasma picture. *Journal of Physics B: Atomic, Molecular and Optical Physics* **2018**, *51*, 09LT01.

(17) Andreasson, J.; Iwan, B.; Andrejczuk, A.; Abreu, E.; Bergh, M.; Caleman, C.; Nelson, A.; Bajt, S.; Chalupsky, J.; Chapman, H.; et al. Saturated ablation in metal hydrides and acceleration of protons and deuterons to keV energies with a soft-x-ray laser. *Physical Review E* **2011**, *83*, 016403.

(18) Hansen, S. B.; Chung, H.-K.; Fontes, C. J.; Ralchenko, Y.; Scott, H.; Stambulchik, E. Review of the 10th Non-LTE code comparison workshop. *High Energy Density Physics* **2020**, *35*, 100693.

(19) Jurek, Z.; Son, S.-K.; Ziaja, B.; Santra, R. XMDYN and XATOM: versatile simulation tools for quantitative modeling of X-ray free-electron laser induced dynamics of matter. *Journal of Applied Crystallography* **2016**, *49*, 1048–1056.

(20) Kozlov, A.; Martin, A. V.; Quiney, H. M. Hybrid Plasma/Molecular-Dynamics Approach for Efficient XFEL Radiation Damage Simulations. *Crystals* **2020**, *10*.

(21) Grånäs, O.; Timneanu, N.; Eliah Dawod, I.; Ragazzon, D.; Trygg, S.; Souvatzis, P.; Edvinsson, T.; Caleman, C. Femtosecond bond breaking and charge dynamics in ultracharged amino acids. *The Journal of Chemical Physics* **2019**, *151*, 144307.

(22) Eliah Dawod, I.; Tîmneanu, N.; Mancuso, A. P.; Caleman, C.; Grånäs, O. Imaging of femtosecond bond breaking and charge dynamics in ultracharged peptides. *Phys. Chem. Chem. Phys.* **2022**, *24*, 1532–1543.

(23) Neese, F.; Wennmohs, F.; Becker, U.; Riplinger, C. The ORCA quantum chemistry program package. *The Journal of Chemical Physics* **2020**, *152*, 224108.
